# Detection of *Toxoplasma gondii* in cat’s internal organs by immunohistochemistry methods labeled with-[strept] avidin-biotin

**DOI:** 10.14202/vetworld.2017.1035-1039

**Published:** 2017-09-04

**Authors:** Muhammad Hanafiah, Raden Wisnu Nurcahyo, Rika Yuniar Siregar, Joko Prastowo, Sri Hartati, Bambang Sutrisno, Dwinna Aliza

**Affiliations:** 1 Parasitology Laboratory, Faculty of Veterinary Medicine, Syiah Kuala University, Banda Aceh, Indonesia; 2 Department of Parasitology, Faculty of Veterinary Medicine, Gadjah Mada University, Yogyakarta, Indonesia; 3 Veterinary Public Health of Laboratory, Diseases Investigation Center, Wates, Yogyakarta, Indonesia; 4 Department of Clinic, Faculty of Veterinary Medicine, Gadjah Mada University, Yogyakarta, Indonesia; 5 Department of Pathology, FKH UGM, Sekip Unit II Yogyakarta 55281, Yogyakarta, Indonesia; 6 Pathology Laboratory, Faculty of Veterinary Medicine, Syiah Kuala University Banda Aceh, Indonesia

**Keywords:** cat, immunohistochemistry, labeled-[strept] avidin-biotin, *Toxoplasma gondii*

## Abstract

**Aim:**

The aims of the study are to detect the presence of *Toxoplasma gondii* antigen and to determine its distribution location in several organs of domestic cat using immunohistochemistry (IHC) method with Labeled-[Strept] Avidin-Biotin (LAB-SA).

**Material and Methods:**

Four domestic cats aged 1-2 years were used as sample in this research. The sample divided into two groups with two cats each. Cats in Group I were positive *Toxoplasma* based on serologically screening test, while cats in Group II were orally infected with 1×10^6^ *Toxoplasma* *oocyst*. All samples then necropsied, and the organs including brain, liver, kidney, duodenum, jejunum, ileum, lungs, and spleen were collected for IHC method with LAB-SA.

**Result:**

The result showed that *Toxoplasma* antigens were detected in ileum of both serologically positive domestic cat and the experimentally infected cats. *Toxoplasma* was also observed in kidney of serologically positive domestic cat. In the serologically positive domestic cat, necrotic lesions were found on ileum, kidney, and liver, whereas in experimentally infected cat, the lesion was only found on ileum.

**Conclusion:**

The presence of *Toxoplasma* antigen is successfully detected in several organs of domestic cat using IHC method with the LAB-SA.

## Introduction

The obligate intracellular parasite *Toxoplasma gondii* infects a broad range of mammalian and avian hosts including approximately one-third of the human population [1-4].

Toxoplasmosis is a globally spread zoonosis with a clinical impact on the unborn fetuses and the immunosuppressed individuals, furthermore, it is regarded as one of the leading causes of death in food-borne illness [5]. Toxoplasmosis is a worldwide reported zoonotic infection caused by the protozoon *Toxoplasma gondii* [6]. *T. gondii* cause a lifelong chronic infection in host by impede and suppress the immune system [7,8].

The domestic and other felids have been proven as the major reservoir host of *T. gondii* infection. It is generally assumed that cats play a major role in transmitting *T. gondii* through fecal contamination of soil, food, and water because they can excrete millions of oocysts in short period of time (1-2 weeks) [9]. The infection can be obtained by eating raw or undercooked meat containing cysts or consuming food or water contained with sporulated oocysts [10,11] or by ingestion of raw milk and milk products [12].

Several laboratory methods have been developed to detect antibody in serum of infected cat such as polymerase chain reaction, enzyme-linked immunosorbent assay, latex agglutination test, indirect hemagglutination test, indirect fluorescent antibody test, and immunochromatography (IC). Although these tests are expensive and require a specialized laboratory, they are more sensitive and specific [13,14].

IC is a rapid, simple, sensitive, and specific diagnostic tool to detect specific antibodies from *T. gondii* infection in cats in field conditions [15]. Fecal flotation technique is used to detect oocyst in fecal samples as well. Although flotations are a reference method for the detection of *T. gondii* oocysts, it has been suggested than an alternative test is also needed due to the microscopic examination is time-consuming and needs an experience microscopist [16].

The study of detecting parasites within host organs could not be conducted easily. To overcome the limitation of conventional methods, new diagnostic methods are devised, such as histopathology and immunochemistry method [17,18]. The most widely used and highly sensitive immunohistochemistry (IHC) method is avidin-biotin method or commonly called avidin-biotin complex (ABC) [19].

The most commonly used avidin-biotin method is labeled avidin-biotin (LAB) or labeled streptavidin avidin-biotin (LSAB) with biotinylated secondary antibody and three reagents from peroxide or alkaline phosphate. This method has higher sensitivity compared to other ABC methods [20].

The purpose of this study is to detect the appearance of *T. gondii* antigen and to determine its distribution location in several organs of domestic cat using IHC method with labeled-(Strept) avidin-biotin (LAB-SA).

## Materials and Methods

### Ethical approval

The study was approved by the Ethics Committee of Ethical Clearance for Pre-clinical Research, Integrated Research and Testing Laboratory, Gadjah Mada University, Yogyakarta, Indonesia (approval no. 00111/09).

### Sample preparation

Four domestic cats aged 1-2 years were used as sample in this research. The sample divided into two groups with two cats each. Cats in Group I were positive *Toxoplasma* based on serologically screening test (tested by Feline Anigen Toxoplasma Ab Rapid Test Kit), while cats in Group II were orally infected with 1×10^6^ *Toxoplasma* *oocyst*. The cats were euthanized using an intravenous injection of 20% pentobarbitone solution as a euthanized material which highly recommended by World Society for the Protection of Animals, London. This method is considered best practice because it consistently produces a death when used as the sole means of euthanasia. Then, a necropsy was performed on both individuals, followed by the collections of the organs including brain, liver, kidney, duodenum, jejunum, ileum, lungs, and spleen for IHC method with LAB-SA (Invitrogen™) process.

### IHC

The LAB-SA test was performed according to the manufacturer instruction (Invitrogen Inc., USA). After deparaffinization, the endogenous peroxidase activity of each tissue was blocked with 0.3% H_2_O. The IHC slides were stained by histostain^®^ (Invitrogen, USA). Four micrometers thick placentome sections were cut from the zinc salt fixed blocks, mounted on positively charged glass slides (Superfrost^®^ Plus, Germany) and air-dried before incubation at 57°C for 1 h. Dewaxing and IHC labeling were then carried out on a Ventana^®^ Benchmark XT (Syntec Scientific Ltd., Ireland) automated IHC staining machine. Slides were incubated with primary polyclonal anti-*T. gondii* antibody (rabbit) primary antibody (Abcam, USA) diluted 1/100 at 37°C for 32 min and biotinylated secondary antibody (Abcam, USA). The primary antibody epitope binding was visualized using the DAB CHROMOGEN (Invitrogen, USA). The sections were counterstained with Mayer hematoxylin and bluing reagent (Invitrogen^®^ LAB-SA Detection System). Citrate buffer (pH 6.0) was used for antigen retrieval and also other reagents from a streptavidin avidin-biotin (LAB-SA) immunoenzymatic antigen.

On completion of staining, slide was raised in a mild detergent solution, and the stained sections were dehydrated through staged alcohols to xylene and coverslipped. Sections were then examined for the presence of positively staining structure consistent with the morphology *T. gondii*. The result interpretation was observed using microscope; positive result showed dark brown tissues (the sample contained antigen), and the negative result showed blue (the sample does not contain the antigen).

### Statistical analysis

The data of antigen *Toxoplasma* examination on cats, both serologically positive and infected toxoplasmosis on organs using IHC were analyzed descriptively.

## Results

### IHC

**Figure-1:**
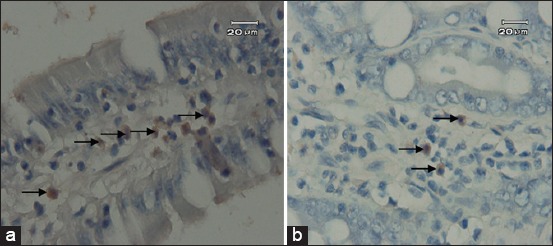
Cat intestine with Toxoplasma positive immunohistochemistry staining. (a) Macrophages in ileum that serologically positive of Toxoplasma (scale bar=20 µm) (arrow); (b) macrophages in the ileum in cats with an experimentally infected cat of Toxoplasma. (Scale bar=20 µm) (arrow).

Results of the examination of several organs by IHC staining are shown in Figure-1a and b:

Macrophages are observed in some organs such as liver, lungs, and kidney of cats which serologically positive *Toxoplasmosis*. Thus, indicating chronically infected. The result than supported by enzyme-linked fluorescent assay (ELFA) examination which positively reacted to immunoglobulin (Ig) G (Immunoserology IgM anti-*Toxoplasma*: Reactive: 8.8 IU/ml). Meanwhile, in cats infected with *Toxoplasma*, macrophages are only found in ileum. This result indicates that the infection is in an acute stage. ELFA examination to strengthened the result was then carried out which showed the positive reactions of all serum to IgM (Immunoserology IgM anti-*Toxoplasma*: Reactive: 0.71 index).

Some clinical symptoms data have been collected from the examination of body temperature, breath, and pulse frequencies. The clinical symptoms in both serologically positive and experimentally infected cat with toxoplasmosis are included increased breathing (ranging between 20-80/min and 41-120/min, respectively) and increased heart beat frequency (81-110 and 110-170 bpm, respectively). The results showed that all samples still in normal standard of healthy cat. However, the clinical data were not specific enough to diagnose cat with toxoplasmosis.

## Discussion

**Figure-2:**
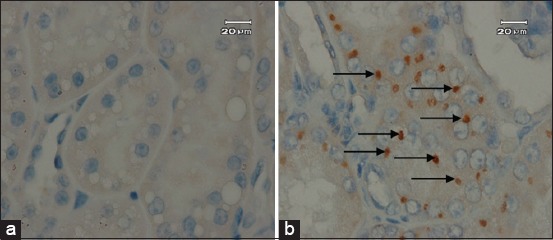
Cat kidney with Toxoplasma positive immunohistochemistry staining. (a) Negative control of immunohistochemistry staining; (b) macrophages in intracytoplasmic tubulus. (scale bar=20 µm) (arrow).

The IHC examination results on several organs of experimentally infected cat show that the only part of the small intestine which showed positive result was the ileum (Figure-1a and b), whereas on the other organs were not found. Immunohistochemistry positive result in serologically *Toxoplasma* positive cat was found intracytoplasmic of epithelial cell of kidney tubules (Figure-2b) and ileum, while on the negative control were not revealed (Figure-2a). Positive reaction was shown by dark brown color, which indicated antigen-antibody binding. The positive reaction in the ileum of both groups existed inside the macrophages.

IHC positive results in this study show a dark brown color. Broad spectrum with diaminobenzidine (DAB) chromogen produces dark brown color on the binding of antigen-antibody *Toxoplasma* on stained tissue. According to Bionisch [19], DAB would block all the residual primary antibodies from the first immune reactions.

Streptavidin biotin (SB) method is faster, sensitive, and clean with strong color intensity, besides there are no color disruptions by the poor quality of the background. The SB method is 4-8 times more sensitive compare to avidin-biotin method, and 8-16 times more sensitive, and accurate than Papanicolaou (PAP) method. In addition, LAB-SA provides superior sensitivity to that of the avidin-biotin complex (ABC) [3,4,7,9]. The LAB-SA method is also sensitive than peroxidase (PAP) methods [3,7] and has the advantage of a single universal labeling reagent (enzyme conjugated streptavidin), instead of specific PAP complexes for each animal system.

The results obtained are almost the same identical to what had been done previously by Ramosvara [20] in a study where all infected tissue of 14 specific-pathogen-free (SPF) cats had been examined histologically for all stages of *Toxoplasma* life cycle. Ileum is the most common sites of infection, although in the case of eight SPF cats the entire small intestine was infected. Infection appeared to be concentrated on the epithelial cells of villi tip. In infected cells, the parasite was found between the core and border.

In this research, the positive IHC reaction was seen by the appearance of macrophage cells in both ileum and kidney. This is because *T. gondii* was phagocytized by macrophage cells, creating the possibility of *T. gondii* antigens to exist in the organ when IHC staining is performed using primary antibody and secondary antibody. According to Hutchison *et al*. [21], Cazabone *et al*. [22] antigen will circulate the parasite products produced at the time the parasites invade macrophages. The presence of this antigen indicating the infection is acute and still active. Antigen circulation, in principle, has the same composition and structure with soluble antigen.

*T. gondii* infection can generate non-specific and specific immunity. Non-specific immunity is conducted by macrophages and natural killer (NK) cells. Specific immunity is proven by the ability of *T. gondii* to induce humoral and cellular response. Cellular response against *T. gondii* infection is more dominant than humoral response. At the initial infection, macrophages infected by *T. gondii* tachyzoites produced interleukin (IL-2), tumor necrotic factor-α, IL-β, and IL-15. Cytokine IL-12 activates NK cells to produce interferon-γ (IFN) [23].

On further infection, when adaptive immunity is activated, tachyzoites that infect macrophages or other cells that act as antigen-presenting cells (APC) was digested by lysosomes. *T. gondii* tachyzoites antigens are then presented by major histocompatibility complex II molecules on the macrophages or APC membrane. The appearance of antigen is recognized and captured by receptors (Th-cell receptor [TCR]) owned by T helper lymphocytes (Th cells). The bond between antigen and TCR triggered Th cells to produce IL-2. *T. gondii* tachyzoites also triggers APC to produce IL-12. IL-12 produced by macrophages and APC working in synergy with IL-2 to cause Th cell differentiation into Th1. Th1 cells produced IFN-γ. Antigen and receptor bond activates CD8 + T cells to produce IFN-γ. The existence of IL-2 produced by Th1 cells also reinforced the activation of CD8 + T cells, resulting in more production of IFN-γ. IFN-γ produced by NK cells, Th1 or CD8 + T encourages macrophages to function as microbicides [23].

Macrophages are the predominant leukocytes beside neutrophil, eosinophil, basophil, monocyte, and lymphocyte [24]. Monocyte in blood circulation will differentiate into macrophage in the tissues. Monocyte or macrophage is the most dominant to be infected by *Toxoplasma* then followed by neutrophil and lymphocyte. The ability of tachyzoites is to reproduce decrease when it infected the neutrophil, but it will increase again when the tachyzoites infected other cells or tissues. All types of cells in various organs, except erythrocytes, can be infected by *T. gondii*. Thus, it is assumed that the cells in the ileum were also infected, which was the reason of macrophage activity increased.

**Figure-3:**
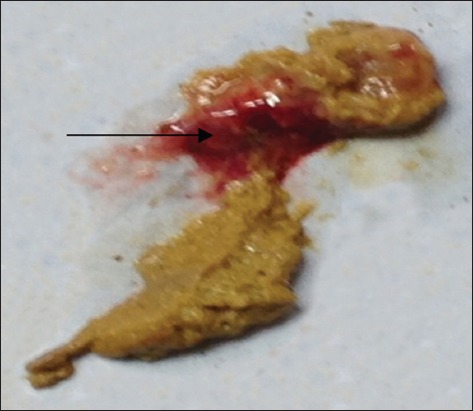
Blood in cat feces which infected by Toxoplasma (arrow).

The presence occurrence of blood (Figure-3) in feces of infected cat is caused by damages on intestinal wall Bañales *et al*. [25] stated that tachyzoites multiply in the lamina propria of the intestine and eventually spread throughout the entire body. Tachyzoites cause tissue damage and eventually develop into bradyzoite stage after which they form tissue cysts.

**Figure-4:**
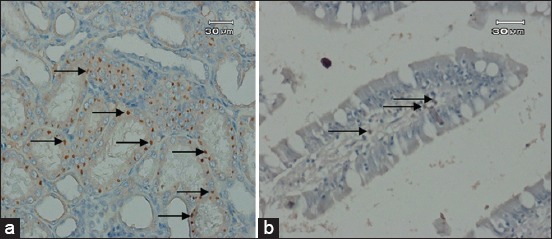
Toxoplasma positive immunohistochemistry staining of cat with labeled-(Strept) avidin-biotin method: (a) Necrosis is present in kidney and (b) ileum (arrow) (brown color is indicating the positive result of immunohistochemistry) (scale bar=30 µm).

Numerous positive lesions were detected in all organs characterized by necrotic lesions (Table-1; Figure-4a and b). All findings are shown in Table-1. Domestic cat serologically *Toxoplasma* positive, necrotics lesions were mainly observed in the ileum, kidney, and liver. While in domestic cat infected by oocyst *Toxoplasma*, the necrotics lesions were mainly observed in the ileum, this caused by the stage of the infection is still preliminary or it is an acute infection, thus the parasite is not spreading to all organs yet. Figure-4 shows the necrosis of the cells of ileum and kidney of cat. The necrosis was in karyolysis category, described by the loss of nucleus in some cells.

**Table-1:** Distribution lesions in cat organ infected by *Toxoplasma.*

Organ	Domestic cat serologically *Toxoplasma* positive		Domestic cat infected by oocyst *Toxoplasma*	
	
	Necrotic lesions	Detection of *T. gondii* by IHC	Necrotic lesions	Detection of *T. gondii* by IHC
Ileum	+	+	+	+
Jejunum	-	-	-	-
Duodenum	-	-	-	
Kidney	+	+	-	-
Lung	-	-	-	-
Liver	+	-	-	-
Spleen	-	-	-	-

IHC=Immunohistochemistry, *T. gondii=Toxoplasma gondii*

## Conclusion

The appearance of *Toxoplasma* antigen is successfully detected in several organs of domestic cat using IHC method with LAB-SA.

## Author’s Contributions

MH and RWN supervised the overall research work. JP, SH, RYS, BS, and DA participated in sampling, made available relevant literatures and executed the experiment and analyzed the tissue and data. All authors interpreted the data, critically revised the manuscript for important intellectual contents and approved the content.
